# The Role of Retinoic Acid in Spermatogenesis and Its Application in Male Reproduction

**DOI:** 10.3390/cells13131092

**Published:** 2024-06-24

**Authors:** Yue Zhao, Shoulong Deng, Chongyang Li, Jingchao Cao, Aowu Wu, Mingming Chen, Xuehai Ma, Sen Wu, Zhengxing Lian

**Affiliations:** 1 Beijing Key Laboratory for Animal Genetic Improvement, National Engineering Laboratory for Animal Breeding, Key Laboratory of Animal Genetics and Breeding of the Ministry of Agriculture, College of Biological Sciences, College of Animal Science and Technology, China Agricultural University, Beijing 100193, China; zhaoyuetx@cau.edu.cn (Y.Z.); chenmingming1937@gmail.com (M.C.); 2 National Center of Technology Innovation for Animal Model, National Health Commission of China (NHC) Key Laboratory of Comparative Medicine, Institute of Laboratory Animal Sciences, Chinese Academy of Medical Sciences and Comparative Medicine Center, Peking Union Medical College, Beijing 100021, China; dengshoulong@cnilas.org; 3 Institute of Animal Sciences (IAS), Chinese Academy of Agricultural Sciences (CAAS), No. 2 Yuanmingyuan Western Road, Haidian District, Beijing 100193, China; 15652652378@163.com; 4 Xinjiang Key Laboratory of Mental Development and Learning Science, College of Psychology, Xinjiang Normal University, Urumqi 830017, China

**Keywords:** retinoic acid, spermatogenesis, male reproductive disorders, male contraception, spermatogonial differentiation, meiosis

## Abstract

Spermatogenesis in mammalian testes is essential for male fertility, ensuring a continuous supply of mature sperm. The testicular microenvironment finely tunes this process, with retinoic acid, an active metabolite of vitamin A, serving a pivotal role. Retinoic acid is critical for various stages, including the differentiation of spermatogonia, meiosis in spermatogenic cells, and the production of mature spermatozoa. Vitamin A deficiency halts spermatogenesis, leading to the degeneration of numerous germ cells, a condition reversible with retinoic acid supplementation. Although retinoic acid can restore fertility in some males with reproductive disorders, it does not work universally. Furthermore, high doses may adversely affect reproduction. The inconsistent outcomes of retinoid treatments in addressing infertility are linked to the incomplete understanding of the molecular mechanisms through which retinoid signaling governs spermatogenesis. In addition to the treatment of male reproductive disorders, the role of retinoic acid in spermatogenesis also provides new ideas for the development of male non-hormone contraceptives. This paper will explore three facets: the synthesis and breakdown of retinoic acid in the testes, its role in spermatogenesis, and its application in male reproduction. Our discussion aims to provide a comprehensive reference for studying the regulatory effects of retinoic acid signaling on spermatogenesis and offer insights into its use in treating male reproductive issues.

## 1. Introduction

Spermatogenesis is a highly organized and sequential process that includes the renewal and differentiation of spermatogonia, the maturation and division of spermatocytes, and the transformation of sperm cells [1]. The process of sperm formation occurs in the seminiferous tubules of the testes. The process begins with undifferentiated spermatogonial stem cells (SSCs) that either continue to self-renew or enter a pathway of differentiation and eventual maturation into spermatozoa [2]. These cells undergo proliferation and differentiation into spermatocytes, which then undergo meiosis to produce spermatocytes. A series of steps culminate in the formation of mature round sperm cells that are released into the lumen of the testicular tubules [3]. Yet, its precise molecular mechanisms remain largely undefined. Retinoic acid (RA) is instrumental in several phases of spermatogenesis. The role of RA in the testis is multifaceted, involving complex intercellular interactions and fine biochemical regulation. Rather than functioning in an isolated environment, RA effectively regulates spermatogenesis through paracrine mechanisms in a complex microenvironment that includes Sertoli cells, mesenchymal cells, and spermatogonia [4]. Specifically, Sertoli cells play a vital role in the development of sperm. They can synthesize RA and release it into the surrounding microenvironment. After that, RA diffuses across the cell membrane and binds to receptors on adjacent spermatogonia. This paracrine action of RA prompts spermatogonia to receive the necessary signals to stimulate their differentiation and enter meiosis. In addition, its ability to resume halted spermatogenic processes due to vitamin A deficiency underscores its crucial role. This paper will systematically detail the entire pathway of RA metabolism and delineate its involvement across different stages of spermatogenesis.

## 2. Overview of Retinoic Acid

Retinoic acid (RA), a metabolic derivative of vitamin A, exists in several isomeric forms, including 9-cis, 13-cis, and all-trans RA. It is crucial for growth and development, playing significant roles in bone growth, epithelial cell proliferation and differentiation, testicular development, and spermatogenesis.

### 2.1. Synthesis of Retinoic Acid

Mammals must obtain vitamin A, the precursor raw material for retinoid synthesis, from the food they consume. As the most significant storage organ of vitamin A in vivo, the liver releases vitamin A and serum retinol-binding protein (RBP4) to form the RBP4-ROL complex, which enters the testes through the blood circulation and then binds to the receptor STRA6 on the cell membrane to achieve the vitamin A transport to the cells for the synthesis of RA [5,6]. Sertoli cells and spermatogenic cells of the testis can synthesize RA required for spermatogenesis [7].

**Figure 1:**
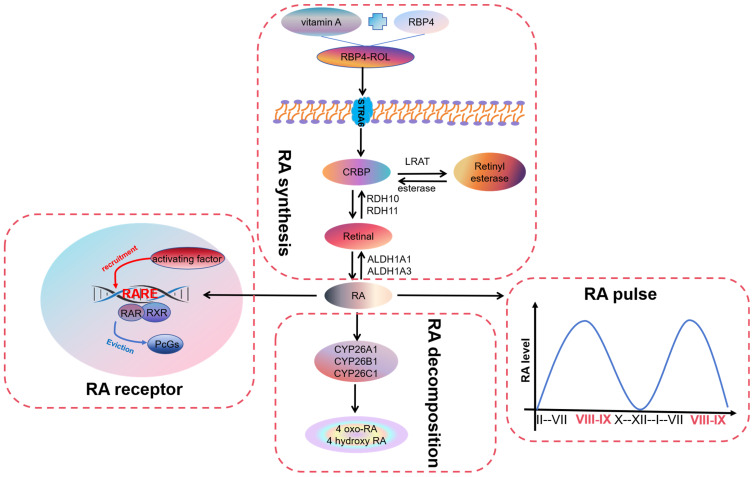
The process of metabolism and function of RA.

Inactive vitamin A must be oxidized to form RA in a two-step enzymatic reaction (Figure 1). The first step of the reaction is reversible, with the oxidation of vitamin A to retinaldehyde catalyzed by two large families of cytosolic alcohol dehydrogenase systems (ADHs) and retinol dehydrogenase systems (RDHs). Three types of ADHs are present in the testis, of which ADH1 and ADH3 are expressed explicitly in Sertoli cells whereas ADH4 is present in late spermatocytes [8,9]. At the same time, the expression of seven different RDHs can be detected in the mouse testis. Among them, RDH10 has been observed in testicular Sertoli cells and spermatogenic cells of prepubertal mice, and this is a key enzyme required for RA synthesis. *RDH10* knockout mice cause embryonic tissue RA deficiency, resulting in embryonic lethality [10]. In addition, at puberty, only undifferentiated spermatogonia are present in most of the seminiferous tubules of the Sertoli cell *RDH10* knockout mice, the lumen is devoid of spermatozoa, and spermatogenesis is severely affected [11]. The second step is irreversible. Retinal is oxidized to RA under the catalysis of retinal dehydrogenase (RALDH). Three RALDHs, RALDH1, RALDH2, and RALDH3 (also known as ALDH1A1, ALDH1A2, and ALDH1A3), can be detected in the testis [12,13]. The specific knockdown of *RALDH2* in germ cells resulted in mice with no abnormalities in spermatogenesis and no reduction in testicular RA levels [14]. However, the deletion of the *ALDH1A1* and *ALDH1A2* genes in the Sertoli cells can completely block the onset of spermatozoa conversion from A spermatogonia to A1, and this blockage can be cured through the injection of RA [15]. It is indicated that the production of RA by Sertoli cells through the ALDH1A1 or ALDH1A2 enzyme is necessary to initiate spermatogenesis. Autophagy-related gene 5 (*Atg5*) also plays a role in RA synthesis. The deletion of *Atg5* in Sertoli cells exacerbated the reduction of RA and ALDH1A1 levels in mouse testis [16]. The 3-Monochloropropane-1,2-diol (3-MCPD) class down-regulates RA synthase ALDH1A1 in primary Sertoli cells [17].

After formation in the cytoplasm, RA is transported to the nucleus, where CRABP2 plays a vital role as a molecular mediator of the biological effects of RA. *CRABP2* is expressed in Sertoli cells and can direct RA to RA receptor-α (RARα) in the nucleus [18]. In addition, CRABP2 may be responsible for activating RA signaling and initiating spermatogonial differentiation [19].

### 2.2. Pulse of Retinoic Acid

The cycle of the seminiferous epithelium is an essential feature of mammalian spermatogenesis [20]. When the proliferating “undifferentiated” spermatogonia enter the differentiation pathway, they enter the cycle. Based on the distribution pattern of spermatozoa in the spermatogenic epithelium, changes in the spermatogonial cell karyotype, meiotic features, and changes in germ cell morphology during development, the mammalian spermatogenic cycle can be divided into twelve phases and four significant clusters of phases (phases I to III, IV to VI, VII to VIII, and IX to XII) [21,22,23]. The time required for differentiation from spermatogonia to sperm is fixed, and this time remains basically unchanged. Thus, when the species is determined, each step in the process of spermatogenesis has a fixed duration, and the germ cells differentiate in strict accordance with the determined time program. In general, the spermatogenic epithelial cycle usually lasts between 8 and 14 days, and the time from the start of spermatogonial differentiation to the formation of mature spermatozoa usually takes more than 40 days. RA is required for the irreversible formation of A1, A2, A3, A4, and B spermatogonia by spermatogonia A. The RA pulse period of adult mice is 8.6 days [24]. RA pulses are produced by Sertoli cells with the help of other testicular cells [25].RA levels were lower in stages II–VI and remained high in stages VIII and IX, with a peak in stage VIII (Figure 1) [26]. The half-life of RA in the mouse testis is approximately 1.3 h. RA signaling in the testis is primarily regulated by ligand concentration (i.e., synthesis and degradation of RA) [27]. Pulses of RA move longitudinally along the tubules, creating a loop in the spermatogenic epithelium. Normal asynchronous spermatogenesis can be modified by altering RA levels, and as a result, the entire testis will consist of a few closely related stages of the cycle. Because the development of germ cells undergoes multiple pulses in the tubules, there are cells in various stages of development throughout the testis at any given time [28]. The RA pulsatile process allows for the continuous release of mature spermatozoa into the lumen of the renal tubules and is essential for sustained fertility. Maintaining RA levels throughout the spermatogenic epithelial cycle is critical for proper spermatogenic regulation [29].

### 2.3. Receptors for Retinoic Acid

In the Sertoli cells and germ cells of the testis, RA functions as a ligand for two significant classes of nuclear receptors, RA receptors (RARs) and retinoid X receptors (RXRs) [30]. RARs and RXRs first bind as heterodimers to the RA response element (RARE) located in the regulatory region of retinoid target genes. In the absence of RA, these heterodimers recruit protein complexes such as polycomb group proteins (PcGs) to down-regulate the transcription of target genes. Following the presence of RA, the receptor’s structure is altered, releasing proteins that inhibit transcription and bind to activators with enzymatic activity, achieving a rise in the expression of downstream target genes (Figure 1) [31]. RAR has three genes encoding isoforms: *RARα*, *RARβ*, and *RARγ*. Similarly, RXR has three genes encoding isoforms: *RXRα*, *RXRβ*, and *RXRγ*. However, other periods or types of cells do not express the identical receptor isoforms, e.g., embryonic rat Sertoli cells express five isoforms. Still, in adulthood, all six isoforms can be expressed [32]. In addition, the same subtype has different effects on different cells. For example, RARα is responsible for the colonization and proliferation of germ cells, but it is responsible for differentiation in Sertoli cells [24,33]. RARα is essential for normal testicular development and RARα-null mice are sterile. Recent studies have found that RARα is required for all its functions in Sertoli cells from puberty onwards. RARα-deficient germ cells can differentiate usually and produce standard live pups [34]. RARβ and RARγ have less of an effect on the process of sperm production, and both RARβ-mutant and RARγ-null males are fertile [35,36]. Of the three isoforms of RXR, RXRβ has been defined as essential for spermatogenesis, with delayed cholesteryl ester accumulation and eventual testicular degeneration in *RXRβ*-null males [37]. *RXRα*-null mice die at embryonic age, and the effect of this isoform on fertility is more difficult to assess [38].

### 2.4. Degradation of Retinoic Acid

The precise degradation of RA is crucial for spermatogenesis. An accurate balance between RA production and degradation in vivo can protect cells from persistent RA signaling, thereby preventing physiological abnormalities. Three cytochrome P450 enzymes (CYP26A1, CYP26B1, and CYP26C1) play a role in RA degradation (Figure 1). Among them, there are many studies on Cyp26b1, which can be expressed in peritubular myoid cells, Sertoli cells, and germ cells and is an essential enzyme in spermatogenesis [39]. Spermatogenic epithelial cells can synthesize and control RA concentrations. CYP26B1 in immature Sertoli cells of the fetal gonads prevents germ cells from entering meiosis and thus inhibits their differentiation [40]. Type A spermatogonia activate NOTCH signaling in Sertoli cells by expressing *JAGGED-1* (*JAG1*), thereby inhibiting the production of CYP26B1. This allows RA to accumulate and activate RA-inducible genes (such as *Stra8*). The effect of germ cell differentiation through changing ligand concentration is achieved [41]. During the embryonic period, CYP26B1 plays a role in the degradation of RA in mouse Sertoli cells. After the deletion of the *CYP26B1* gene, the undifferentiated state of male germ cells was broken and germ cell apoptosis was caused [42,43]. Similarly, treatment with CYP26 enzyme inhibitors increased the expression of reproductive-cell differentiation markers stimulated by RA gene 8 (*Stra8*) and kit oncogene (*Kit*) and a decrease in the expression of POU class 5 homeobox 1 (*Pou5f1*), which initiates spermatogonial differentiation [44]. The knockout of *CYP26B1* in spermatogonia or Sertoli cells only causes mild sperm damage. Only the simultaneous knockout of *CYP26B1* in spermatogonia and Sertoli cells can cause significant sperm damage, resulting in reduced fertility [39]. 

In addition to the cytochrome P450 enzymes, CRABP1 of the cellular RA-binding protein (CRABP) family has been suggested to have a role in targeting the degradation of RA and maintaining spermatogonia in an undifferentiated state. CRABP1 is a cytoplasmic protein expressed only in spermatogonia and not in other germ cells and Sertoli cells, and this unique positional distribution has been associated with preventing vincristine receptor activation [45,46]. After gonadotropin stimulation, the expression of *CRABP1* increased, but the expression of *CRABP11* decreased [19]. CRABP1 isolates RA in the cytoplasm, thereby weakening RA signal transduction. In addition, CRABP1 affects the expression of genes related to RA biosynthesis, transport, and metabolism [47].

After entering the cell, vitamin A in the blood is oxidized to RA by a two-step enzymatic reaction. RA in the testis is released in the form of pulses and regulates the expression of downstream genes by binding to RAR/RXR. Finally, it is mainly degraded by cytochrome P450 enzymes and loses activity.

## 3. Retinoic Acid in Spermatogenesis

RA regulates multiple stages of spermatogenesis, including spermatogonial differentiation, spermatocyte meiosis, and spermatogenesis [29,48]. It plays a critical regulatory role in spermatogenesis, and its regulatory mechanisms and pathways of influence are essential for understanding the molecular basis of spermatogenesis, especially at the stages of spermatogonial differentiation and sperm maturation.

### 3.1. Retinoic Acid in the Differentiation of Spermatogonia

RA is an essential molecule in the differentiation of spermatogenic cells and promotes the transformation of undifferentiated spermatogonia to differentiated spermatogonia [49,50,51]. The process of spermatogonial differentiation involves multiple biological stages and complex molecular regulatory mechanisms in which RA plays a key role. When the RA signal is damaged, the differentiation of spermatogonia is inhibited [52]. RA stimulates the PI3K/Akt/mTOR kinase signaling pathway and promotes spermatogonial differentiation [53]. Since the transformation process of undifferentiated spermatogonia to A1 spermatogonia requires the involvement of RA, the absence of RA will lead to an increase in the number of spermatogonial stem cells in the mouse testis [54]. In mice fed long-term vitamin-A-deficient diets or given an RA synthase inhibitor such as WIN18446, spermatogonia could not pass the Aal stage and remain in the undifferentiated stage, resulting in no spermatogenesis and infertility. In contrast, supplementation with vitamin A or RA enables spermatogonia to complete the transformation from Aal to the A1 type and restore their fertility [55]. RA signaling regulates spermatogonial differentiation by modulating the expression of its direct target genes. In Sertoli cells, RA induces the expression of Kit gene encoding KL receptor (*KL*) and bone morphogenetic protein 4 *(BMP4*) and inhibits the expression of glial cell line-derived neurotrophic factor (*GDNF*) [56]. In undifferentiated spermatogonia, RA binds to RARG to form a complex, which induces the expression of *Kit* and *Stra8* genes [51,57]. NANOS2, an RNA-binding protein essential for maintaining the function and survival of undifferentiated spermatogonia, silences *stra8*, a gene involved in spermatogonial differentiation and entry into meiosis [58]. Thus, RA can promote spermatogonial differentiation by inhibiting NANOS2. In addition, RA induced the differentiation of cultured undifferentiated spermatogonia to form differentiated spermatogonia in vitro [59]. At present, many studies have reported the method of using RA to induce the differentiation of SSCs in vitro [60]. However, studies on the regulatory mechanisms of RA during spermatogenesis are still unclear.

**Figure 2:**
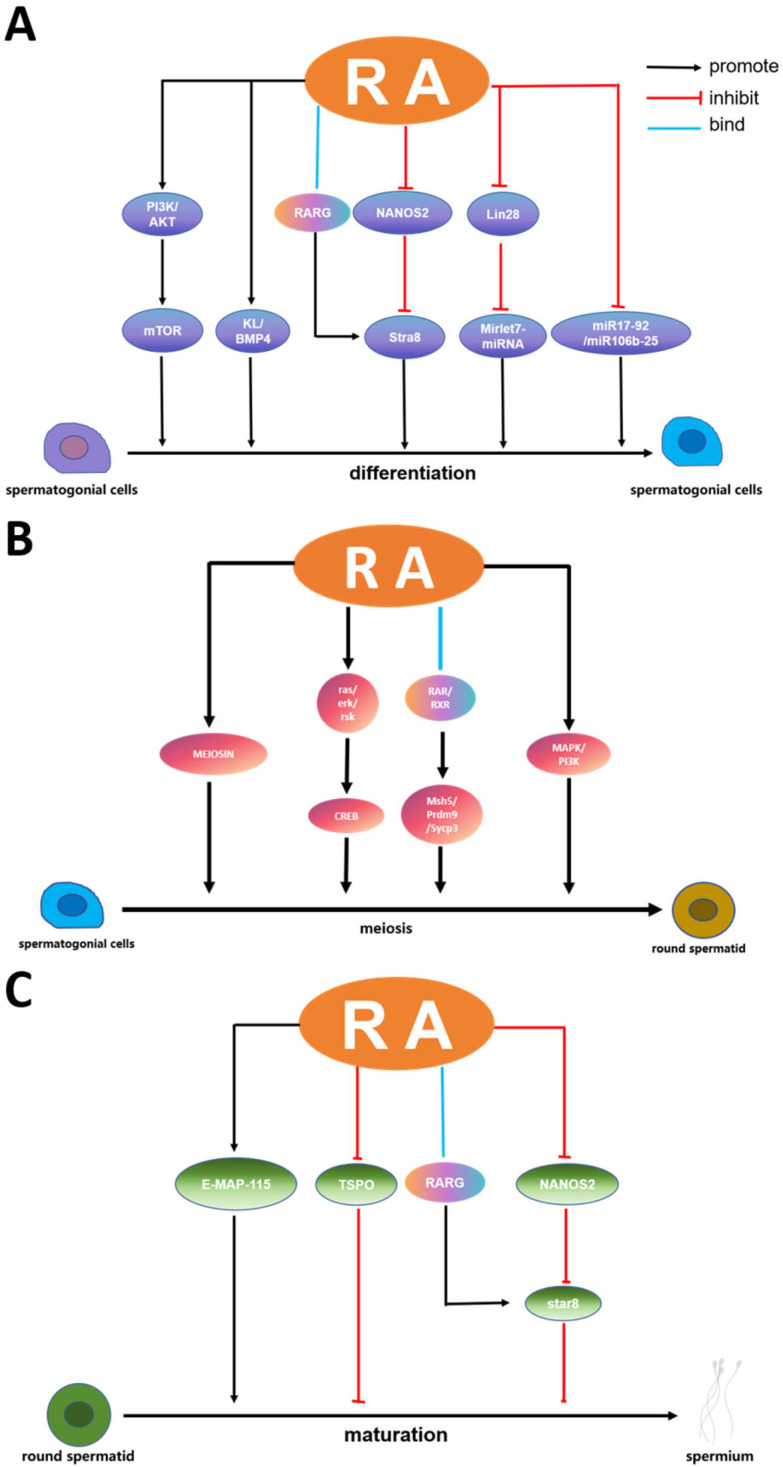
RA regulates spermatogenesis. (**A**): at the differentiation stage. RA can stimulate the PI3K/Akt/mTOR kinase signaling pathway [53]. RA can also induce *KL* and *BMP4* expression to promote differentiation [56]. RA binds to RARG and further induces the expression of *Kit* and *Stra8* genes [51,57]. NANOS2 can silence Stra8, a gene involved in spermatogonial differentiation and meiosis, and RA can promote spermatogonial differentiation by inhibiting NANOS2 [58]. RA promoted spermatogonial differentiation by inhibiting Lin28-induced Mirlet7-miRNA expression [61,62]. RA can also down-regulate the members of miR-17-92 and miR-106b-25 clusters [63]. (**B**): at the meiosis stage. RA directly stimulates Kit phosphorylation to make it active, which regulates DNA synthesis in mitosis and spermatogonia into meiosis through MAPK and PI3K signaling pathways [65]. During meiosis, MEIOSIN is also a downstream target of RA, and RA can activate MEIOSIN expression [66]. RA activates key genes involved in meiosis through its receptors RARs and RXRs, such as *Msh5*, *Prdm9* and *Sycp3* [67,68]. RA can also rapidly activate protein kinase c and transmit the activation signal to CREB through the ras/erk/rsk pathway without the pathway of RA-related receptors [69]. (**C**): at the formation and release stage. RA can participate in the process of sperm release by regulating the protein E-MAP-115 [70]. Similar to the differentiation stage, RA can also stimulate the expression of Stra8 in spermatogonia and reduce the expression of Nanos2 [71,72]. In addition, *TSPO* expression was significantly reduced during RA-induced differentiation [73].

As well as directly regulating gene expression, RA further controls the process of spermatogonial differentiation by affecting regulatory molecules such as microRNAs and RNA-binding proteins. These molecules can regulate the post-transcriptional modification and expression of RA target genes and accurately regulate the behavior and fate of spermatogonia. For example, microRNAs can target and regulate the mRNA stability and translation efficiency of multiple key genes, affecting the expression and function of crucial proteins such as *Stra8*, *Sohlh1*, *Sohlh2*, and *Kit* [39]. The expression of these genes promotes spermatogonia to enter the meiosis process and eventually form sperm. RA promotes spermatogonial differentiation by inhibiting Lin28-induced Mirlet7-miRNA expression [61,62]. RA-induced spermatogonial differentiation is significantly down-regulated in members of the miR-17-92 (Mirc1) and miR-106b-25 (Mirc3) clusters, concomitant with the up-regulated expression of Bim (*Bcl2l11*), *Kit*, *Socs3,* and *Stat3*. Male germ cell-specific miR-17-92 (Mirc1) knockout mice had smaller testes, fewer epididymal spermatozoa, and mild spermatogenesis defects [63]. Some members of the miR-17-92 cluster, such as miR-17, miR-18a, and miR-20a, may all play key roles in spermatogenesis. For example, miR-17 is highly expressed in the early stage in germ cells and is significantly reduced as germ cells mature while miR-20a is mainly present in spermatogonia and primary spermatocytes (Figure 2A). Furthermore, gga-miR-31 transcription is positively regulated by RA and competes with Stra8 to bind RA [64]. RA can be used to synchronize the sperm formation process and the differentiation of spermatogonia populations by controlling the RA supply. The synchronized differentiation of spermatogonia populations can be achieved by blocking RA synthesis with WIN18446 and exogenously supplementing RA at specific time points [48]. This synchronous differentiation not only helps study the response of spermatogonia to RA signals but also facilitates the separation and study of cells at different stages of spermatogenesis.

### 3.2. Retinoic Acid in the Initiation of Meiosis in Spermatogenic Cells

RA is also a key regulator for spermatogonial differentiation and for their entry into meiosis [74]. RA participates in this process through multiple mechanisms, especially playing a central role in initiating meiosis in spermatogonia. It has been shown that a concentration of 10^−8^ mol/L RA can activate *Stra8* to promote meiotic initiation. In *Stra8*-null mice, undifferentiated spermatogonia accumulated abnormally high within 10 days after birth while differentiated spermatogonia were depleted [75]. Doublesex-related transcription factor 1 (DMRT1) blocks meiosis by directly inhibiting RA and indirectly inhibiting *Stra8*. At the same time, DMRT1 also contributes to an increase in the transcriptional activity of the *Sohlh1* gene to hinder the onset of meiosis and thus promote spermatogonial maturation [76,77]. The specific knockdown of the RA-synthesis key enzyme genes *RDH10* or *ALDH1A1~3* in intratesticular cells of the testis resulted in a consequent lack of RA in the mouse testis, causing spermatogenesis to stagnate at the undifferentiated spermatogonia stage [11]. Promyelocytic leukemia zinc-finger (PLZF) maintains SSC in an undifferentiated state, and RA triggers spermatogonial differentiation by directly or indirectly down-regulating *PLZF* [78]. RA directly stimulates the phosphorylation of Kit to make it active, and Kit regulates DNA synthesis in mitosis and the entry of spermatogonia into meiosis through the MAPK and PI3K signaling pathways [65].

The interaction between RA and follicle-stimulating hormone (FSH) constitutes an important regulatory axis during spermatogenesis [44]. FSH is a hormone secreted by the pituitary gland, which promotes sperm production by stimulating Sertoli cells in the testis in the reproductive system. Without FSH, the proliferation of spermatogonia and their transformation into spermatocytes will be inhibited [79,80]. FSH converts retinal into RA by regulating the expression of enzymes such as ALDH1A2 [81]. This regulatory mechanism allows FSH to indirectly control RA, thereby affecting the maturation and differentiation of spermatogonia. In addition, FSH up-regulates RARα expression to enhance the sensitivity of spermatogonia and Sertoli cells to RA, making these cells more responsive to RA and promoting the initiation of meiosis and subsequent stages of spermatogenesis [82]. FSH, as well as directly activating vincristine synthesis in Sertoli cells, also involves regulating *GFRα1* expression in such cells, affecting spermatogonia self-renewal and partitioning.

### 3.3. Retinoic Acid in the Late Meiotic Process in Spermatogenic Cells

During late meiosis in spermatogenic cells, chromosomes undergo homologous chromosome segregation while sister chromatid monomers are segregated into different daughter cells, ultimately forming haploid spermatocytes. This process is essential for spermatogenesis and occurs intact in the mammalian testicular spermatogenic epithelium. RA facilitates this process by regulating the expression of critical genes, which play an important role.

At late meiotic stages in spermatogenic cells, RA activates vital genes involved in the meiotic process, such as *Msh5*, *Prdm9*, and *Sycp3*, via their receptors RARs and RXRs [83]. The proteins encoded by these genes play essential roles in late meiosis, with *Msh5* involved in chromosome pairing and recombination, *Prdm9* in chromosome recombination exchange, and *Sycp3* in chromosome pairing and recombination [67,68]. In terms of transcriptional regulation, RA can rapidly activate protein kinase c and transmit the activation signal to CREB through the ras/erk/rsk pathway without the pathway of RA-related receptors [69]. cAMP acts during the activation phase of spermatogenesis on meiosis-related genes (*Prm* and *Tnp*), and many meiosis-related gene sequences contain CREB [84]. In particular, the cAMP-responsive element modulator (CREM) of the CREB family plays an important role in regulating transcriptional activation in spermatogenesis, especially in late meiotic germ cells (Figure 2B) [85]. CREM inactivation results in an increased rate of apoptosis at the round spermatocyte stage. At the same time, the expression of apoptosis-related genes is elevated in CREM knockout mice [86]. During embryo differentiation and formation, active CREB proteins increased significantly after treatment with 5 μmol/L RA [87].

In meiosis, MEIOSIN is also a downstream target of RA [66]. It is an important meiotic protein encoded by the *Gm4969* gene that can act synergistically with STRA8 to regulate meiotic timing and initiation [88]. It should be noted that MEIOSIN is only present in spermatogonia at stages VIII–IX in the presence of RA and is not expressed in spermatogonia at the same stage of the spermatogenic epithelium. WIN18446 is a potent inhibitor of RA activity in the testis, acting directly by inhibiting retinoid dehydrogenase and subsequently blocking RA production [89,90]. WIN18446, a potent inhibitor of intratesticular RA activity, also inhibits MEIOSIN expression, while the administration of exogenous RA reactivates MEIOSIN expression. In the clinical arena, the inhibition of RA activity by WIN18446 could serve as a new target for male contraceptives.

### 3.4. Retinoic Acid in the Formation and Release of Spermatozoa

In spermatogenesis, RA contributes to the production and release of spermatozoa in the testicular seminiferous tubules. RA is not only involved in spermatogenesis but can also affect sperm release. For example, in Vitamin A deficiency (VAD), spermatozoa are retained in the spermatogenic epithelium and cannot be released generally into the lumen of the seminiferous tubules [91]. Similarly, spermatozoa from *Rbp4* and *Lrat* knockout mice fed a VAD diet show the same phenomenon [36,92]. This failure to release sperm typically has also been seen in *RARα* and *RARβ* knockout mice [93]. RA can be involved in the process of sperm release in mice by modulating the protein E-MAP-115 [70]. RA has also stimulated *Stra8* expression in spermatogonia while decreasing *NANOS2* expression [71,72]. The Stra8 and Setd8 ubiquitination degradation pathways regulate spermatogenesis and protect against spermatids [94]. In addition, RA supported androgen-induced meiosis and reduced apoptosis in spermatogonia and spermatocytes [44,95]. Translocator protein (TSPO) is present at specific spermatogenesis stages such as the formation of round sperm acrosomes. It acts as an inhibitor of differentiation, and *TSPO* expression is significantly reduced during RA-induced differentiation (Figure 2C) [73].

In addition to the direct regulation of downstream genes by RA to influence sperm formation and release, the receptor expression of RA has also been closely linked to sperm formation and release. RARα has roles in meiosis, sperm maturation, and the development of testicular Sertoli cells [96]. RARα changes the morphology of sperm by regulating the expression of related genes. The first spermatogenesis wave in *RARα* knockout mice is blocked at the spermatocyte 8 stage. After the specific overexpression of RARα, spermatogenesis returned to normal in these mice [97]. RAR antagonist BMS-189453-treated mice have impaired spermatogenesis, including abnormal spermatocyte orientation, translocation, and release [98]. The mRNA expression levels of *RARα*, *RARγ*, *RXRα*, *RXRβ*, and *RXRγ* are significantly decreased in patients with Sertoli cell syndrome (SCO) and maturation arrest (MA). The subcellular localization of RARα, RARγ, RXRβ, and RXRγ in HYPO patients was not significantly different from that in normal males. This finding suggests that decreased mRNA expression levels of *RARα*, *RARγ*, *RXRα*, *RXRβ*, and *RXRγ* are more associated with the development of SCO and MA types of oligoasthenozoospermia than with the development of oligoasthenozoospermia in which cell numbers are reduced [99].

## 4. The Application of Retinoic Acid in Male Reproduction

### 4.1. The Use of Retinoic Acid in the Treatment of Male Reproductive Disorders

Male factor contributes to ~50% of infertile cases worldwide, involving 50 to 80 million men. There are many causes of primary male infertility, including azoospermia, oligospermia, hypospermia, and spermatozoa incompetence. A decrease in sperm count or concentration increases the incidence of male infertility. Therefore, the decline in testicular sperm count remains a concern. RA plays an important role in spermatogenesis, and its association with infertility is of interest.

RA has shown positive therapeutic effects in a number of male infertility conditions. Cryptorchidism, a congenital malformation type of disorder, can affect male fertility [100]. Patients with cryptorchidism have a significant reduction of germ cells in the testes, and even sperm can disappear completely. Spermatogonia isolated from cryptorchid patients are co-cultured with RA and stem cell factor (SCF), and the spermatogonia begin to differentiate and eventually form spermatozoa [101]. In addition, RA concentration and RA gene 8 (*Stra8*) expression are significantly lower in mice with cryptorchidism than in normal controls. Therefore, RA supplementation or up-regulation of Stra8 may offer new hope for the treatment of cryptorchid spermatogenesis [102,103]. In addition to cryptorchidism, there is a potential therapeutic effect of RA on varicocele. The incidence of varicocele is as high as 10–15 percent. Reactive oxygen species (ROS) are significantly increased in the semen of patients with varicocele; in addition, the level of molecular expression of RAR-A protein is reduced in the sperm cells of patients with varicocele [104]. The reduced production of malondialdehyde and reactive oxygen species (ROS) in spermatozoa after treatment with RA protects spermatozoa from reactive oxygen species (ROS) damage [105]. Some infertile patients are characterized by significantly reduced testicular-tissue ALDH1A2 protein levels, and their sperm counts have improved with oral retinoids [106]. Nineteen men were selected who had been infertile for more than 12 months and whose sperm concentration was less than 15 million sperm/mL. In all men who received 20 mg of oral isotretinoin twice daily for 20 weeks, sperm concentration increased from 2.5 million/mL to 3.8 million/mL at the end of treatment, with a significant improvement in sperm morphology [106]. The above indicates that RA has potential therapeutic effects on male infertility. Because of the characteristics of RA, such as low cost and toxicity, it has great advantages in the future clinical therapeutic field.

Although the toxicity of RA is low, it should not be used indiscriminately. High doses of RA can negatively affect animal reproduction. Excessive doses of RA have been shown to cause testicular degeneration or impaired spermatogenesis in mice, lizards, and dogs [107]. In addition, it has been shown that after excessive RA treatment, fetal testicular development is impaired, the number of germ cells is reduced, the ecological niche of somatic cells is altered, and the structure of the spermatogenic ridge is impaired. Excessive RA in the testes disrupts normal meiotic regulation, which can lead to the development of testicular dysgenesis [108]. Excessive RA may lead to malformations in different organ systems, and germ cell dysplasia in idiopathic non-obstructive azoospermia (iNOA) correlates with increased RA signaling in Sertoli cells [109]. In summary, the molecular mechanism of RA in regulating spermatogenesis needs to be further explored to conclude that those infertility conditions are suitable for RA treatment and to provide new possibilities for the treatment of infertility.

### 4.2. The Use of Retinoic Acid in Male Contraception

Nearly half of pregnancies worldwide are unplanned. In the United States alone, unwanted pregnancy has caused billions of dollars in losses every year [110]. In order to reduce the rate of unintended pregnancy, researchers have focused on the development of male contraceptives [111]. Because of the role of RA in spermatogenesis, the development of non-hormone male contraceptives for the mechanism of RA has received extensive attention [112]. At present, the research and development strategies of contraceptives for RA mainly focus on two aspects: inhibitors of RA synthesis and antagonists of RAR [113]. More than 60 years ago, rats treated with RA inhibitors were found to be infertile. Similarly, the oral dosing of WIN18446, which inhibits testicular RA biosynthesis, effectively contracepts rabbits [114]. Thus, RA inhibitors have become potential non-hormone male contraceptives [115]. WIN18446 can inhibit ALDH1A1 and ALDH1A2, inhibiting RA biosynthesis in the testis [116]. In 60 male subjects, WIN18446 rendered them infertile for 1 year. However, male subjects treated with WIN18446 experienced severe disulfiram reactions after drinking alcohol, with symptoms such as vomiting, sweating, and palpitations. Due to the disulfiram reaction, WIN18446 can no longer be used as a male contraceptive. At present, a large number of studies have focused on the development of RAR antagonists, and a variety of drugs that may become male contraceptives have been discovered. BMS-189453, a pan-RA receptor antagonist that acts on α, β, and γ receptors, was reported about 30 years ago [117]. In mice, BMS-189453 reversibly inhibited spermatogenesis. In the rat model, the use of low-dose BMS-189453 can cause rats to lose fertility. They can regain fertility in a reversible manner upon the drug withdrawal [118]. However, doses above 240 mg/kg can cause severe liver poisoning and death [98]. YCT-529 was reported as a potential male contraceptive at the 2022 National Conference of the American Chemical Society [119]. When given to mice orally for 4 weeks, it reduced sperm counts and achieved 99% of pregnancy. All the mice regained fertility. As a male contraceptive pill, YCT-529 entered a phase 1 clinical trial in the UK on 13 December 2023, which was an important advance in the field of contraception. In addition to the above drugs, scientists have also developed BMS-189532, BMS-189614, Chromene amide analogue 21, and ER-50891. Whether these drugs can be used as male contraceptives may need further evaluation [120,121,122,123]. In general, the development process of RA inhibitors as non-hormone male contraceptives is complex, but it has a good prospect.

## 5. Summary

RA has a significant role in spermatogenesis. We introduced, in detail, the metabolic process of RA in the testis and systematically described its role in multiple stages of spermatogenesis. However, more deeply, the related pathways of RA regulating spermatogenesis have not been fully explored. At the same time, this paper has summarized some applications of RA in male reproduction. To further examine the role of RA in male contraception and the treatment of male reproductive disorders, a clear regulatory network of RA regulating spermatogenesis is indispensable. We believe that by summarizing the existing work, we can provide some help in parsing the mechanisms related to the regulation of spermatogenesis by RA.
